# RNA-Seq-Based Comparative Transcriptome Analysis Highlights New Features of the Heat-Stress Response in the Extremophilic Bacterium *Deinococcus radiodurans*

**DOI:** 10.3390/ijms20225603

**Published:** 2019-11-09

**Authors:** Dong Xue, Wenzheng Liu, Yun Chen, Yingying Liu, Jiahui Han, Xiuxiu Geng, Jiang Li, Shijie Jiang, Zhengfu Zhou, Wei Zhang, Ming Chen, Min Lin, Marc Ongena, Jin Wang

**Affiliations:** 1 Biotechnology Research Institute, Chinese Academy of Agricultural Sciences, Beijing 100081, China; xue_dong_kevin@126.com (D.X.); chenyun0402ye@163.com (Y.C.); liuyingying03@126.com (Y.L.); 13121257599@163.com (J.H.); zhouzhengfu@caas.cn (Z.Z.); zhangwei01@caas.cn (W.Z.); chenming01@caas.cn (M.C.); linmin57@vip.163.com (M.L.); 2 Microbial Processes and Interactions (MiPI), TERRA Teaching and Research Centre, Gembloux Agro-Bio Tech, University of Liège, 5030 Gembloux, Belgium; 3 School of Food and Pharmaceutical Engineering, Nanjing Normal University, Nanjing 210023, China; wzliuouc@gmail.com; 4 Department of Plant Science, School of Agriculture and Biology, Shanghai Jiao Tong University, Shanghai 200240, China; 5 College of Life Science and Engineering, Southwest University of Science and Technology, Mianyang 621000, China; shijiejiang525@163.com

**Keywords:** *Deinococcus radiodurans*, recovery, RNA-Seq, heat stress, novel heat-related gene, cell wall damage

## Abstract

*Deinococcus radiodurans* is best known for its extraordinary resistance to diverse environmental stress factors, such as ionizing radiation, ultraviolet (UV) irradiation, desiccation, oxidation, and high temperatures. The heat response of this bacterium is considered to be due to a classical, stress-induced regulatory system that is characterized by extensive transcriptional reprogramming. In this study, we investigated the key functional genes involved in heat stress that were expressed and accumulated in cells (R48) following heat treatment at 48 °C for 2 h. Considering that protein degradation is a time-consuming bioprocess, we predicted that to maintain cellular homeostasis, the expression of the key functional proteins would be significantly decreased in cells (RH) that had partly recovered from heat stress relative to their expression in cells (R30) grown under control conditions. Comparative transcriptomics identified 15 genes that were significantly downregulated in RH relative to R30, seven of which had previously been characterized to be heat shock proteins. Among these genes, three hypothetical genes (*dr_0127*, *dr_1083*, and *dr_1325*) are highly likely to be involved in response to heat stress. Survival analysis of mutant strains lacking DR_0127 (a DNA-binding protein), DR_1325 (an endopeptidase-like protein), and DR_1083 (a hypothetical protein) showed a reduction in heat tolerance compared to the wild-type strain. These results suggest that DR_0127, DR_1083, and DR_1325 might play roles in the heat stress response. Overall, the results of this study provide deeper insights into the transcriptional regulation of the heat response in *D. radiodurans*.

## 1. Introduction

*Deinococcus radiodurans* (*D. radiodurans*) is a nonmotile, pink-pigmented, Gram-positive bacterium belonging to the Deinococcus-Thermus phylum. This bacterium was first isolated from gamma-irradiated canned meat and is best known for its extraordinary resistance to ionizing and ultraviolet (UV) irradiation [1,2]. Previous studies have reported that this species presents a rapid response and adaptation to a wide variety of extreme environments and stresses, such as desiccation [3], hydrogen peroxide [4], heat [5], and other abiotic stresses. The rapid adaption of *D. radiodurans* is accompanied by a powerful DNA repair ability and extreme stress resistance. Hence, this species has become an ideal model organism for studying bacterial tolerance mechanisms under various extreme stress conditions [6,7].

One of the earliest studies conducted on heat stress in *D. radiodurans* suggested that proteins synthesized de novo during the incubation interval were involved in either the appearance of thermotolerance or in the recovery from injury induced by heating at 52 °C for 30 min [8]. In a later work, sigma factors (*sig1* and *sig2*) were identified as being involved in the active response of *D. radiodurans* to high-temperature stress. The *sig1* gene was identified as essential for the induction of the heat shock proteins GroESL and DnaKJ, whereas a *sig2* mutant exhibited only modest deficiencies in DnaKJ production [9,10]. The global negative regulator encoded by *dr_0934* (HspR) binds to HspR-associated inverted repeat (HAIR) sites in close proximity to promoter regions, thereby directly inhibiting the expression of regulated genes encoding chaperone proteins and protease [11]. 2D-PAGE and global whole-cell Fourier transform ion cyclotron resonance mass spectrometric (FTICR-MS) proteomics have been widely employed to identify the molecular mechanisms underlying the heat tolerance of *D. radiodurans* at the proteomics level. The majority of the highly-induced heat shock proteins were identified by matrix-assisted laser sesorption/ionization mass spectrometry (MALDI-MS) [12,13]. The results of a more recent report investigating the DNA repair capacity and membrane integrity of *D. radiodurans* under dry and wet heat suggest that DNA damage repair (e.g., DNA double-strand breaks by *recA* and *pprA*) is essential after treatment with wet or dry heat [14]. Two small heat shock proteins (sHsps) identified in *Escherichia coil*, IbpA and IbpB, were analyzed in *D. radiodurans*, with subsequent analyses showing that these two proteins were very different in their quaternary structures and chaperone properties and were considered to represent a second type of bacterial two-component sHsp system [15]. Recently, DdrI (encoded by *dr_0997*) has been reported to be involved in the heat shock response [16].

Heat treatment has been reported to result in protein unfolding and aggregation in microbes [17,18,19]. Accordingly, microorganisms have evolved complex molecular mechanisms to mitigate heat stress [17,18,20,21]. Previous studies have shown that bacteria display both common and unique changes in their gene expression profiles in response to temperature fluctuations in their surrounding microenvironments [22,23,24]. Most heat stress-induced genes encode molecular chaperones or proteases that either protect proteins/enzymes from misfolding or accelerate the degradation of damaged proteins. These processes lead to changes in the cell wall/membrane composition, protein synthesis rates, energy metabolism, and other biological processes, thereby maintaining internal cell homeostasis [18,19,21,25,26,27]. The use of RNA sequencing (RNA-Seq) allowed Chan et al. (2016) and Gomide et al. (2018) to identify many unanticipated genes associated with heat tolerance in *Pseudomonas aeruginosa* and *Corynebacterium pseudotuberculosis* following heat shock [28,29].

Although much effort has been put into elucidating the molecular mechanisms underlying the response of *D. radiodurans* to heat stress, gene expression in *D. radiodurans* during heat stress is complex and not fully understood. However, due to the rapid development of sequencing technologies and bioinformatic analysis, many novel functional genes have been identified in *D. radiodurans* under a variety of stresses [30,31,32]. RNA-Seq has been successfully used to determine the deep transcriptional profiles of a complete genome in specific environments [33,34,35]. In this study, we utilized an RNA-Seq-based technique to examine the changes in the transcriptome of *D. radiodurans* in response to heat stress. Under heat stress, heat-related proteins are highly expressed to cope with cell metabolism and protein changes. When the external environment is restored to conditions that suitable for growth, a large amount of heat stress-related proteins will accumulate. Considering that degradation of these protein is a time-consuming bioprocess, we predicted that to maintain cellular homeostasis, the expression of key functional proteins would be significantly decreased in cells (RH) that had partly recovered from heat stress relative to their expression in cells (R30) grown under control conditions. Through this analysis, we identified many of the classical heat shock genes, the expressions of which were significantly increased in response to elevated temperature. In addition, our results show that three hypothetical genes (*dr_0127*, *dr_1083*, and *dr_1325*) might play important roles in the heat stress adaptation through mechanisms that require future study. The results of this study provide insight into the transcriptional regulation of the heat tolerance in *D. radiodurans*.

## 2. Results

### 2.1. Cell Growth State and Viability of D. radiodurans under Heat Stress

**Figure 1:**
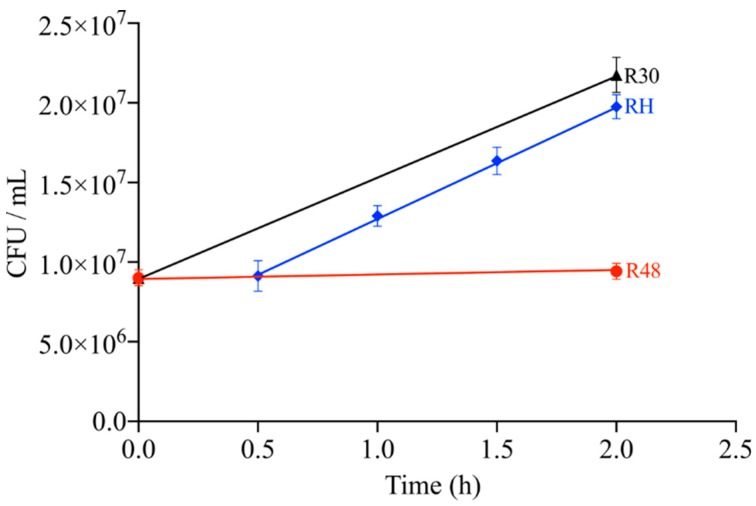
Correlation analysis of the cell viability and the different treatments using three biological replicates. The triangle symbol with the black line represents the untreated group (R30), the circles with the red line represent the heat treatment group (R48), and the diamond dots with the blue line represent the recovery treatment group at various incubation periods (RH). Error bars represent the SD calculated from three sets of independent experiments.

In order to investigat the mechanism of adaptation to heat stress of *D. radiodurans*, we used a series of recovery times (0, 0.5, 1, 1.5, and 2 h) for growth at 30 °C following incubation at 48 °C. This series allowed us to determine the time necessary for the cells to return to their initial growth state after the heat stress treatment. We found that after 2 h of cell recovery, the growth viability according to the cell number was essentially the same as that of R30 subjected to continuous culture for 2 h (Figure 1).

**Figure 2:**
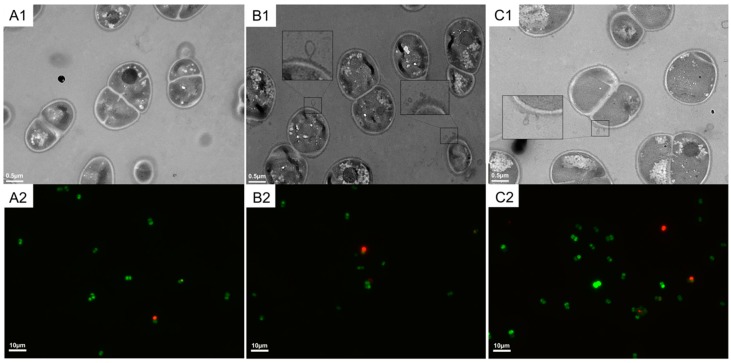
TEM and fluorescence images of the heat-induced lesions on *Deinococcus radiodurans* cells following exposure to 48 °C for 2 h. (**A1**,**B1**,**C1**) represent the TEM results; (**A2**,**B2**,**C2**) represent the fluorescence analysis results. (**A1**,**A2**) *D. radiodurans* cells at 30 °C (control samples), (**B1**,**B2**) *D. radiodurans* cells at 48 °C (heat-treated samples), and (**C1**,**C2**) *D. radiodurans* cells recovered at 30 °C after the heat treatment (recovery samples). The inset diagram (**B1**,**C1**) show an amplified region of the cell envelope. Living cells were stained by Redox Sensor Green (RSG) (green), and dead cells were stained by propidium iodide (PI) (red). The scale bars indicate the corresponding lengths.

Because heat stress may affect the integrity of *D. radiodurans*, we used transmission electron microscopy (TEM) to observe the morphology of *D. radiodurans* cells. As shown in Figure 2A1,B1, the ultrastructure of cells incubated at 48 °C (R48) was different compared to R30, and R48 exhibited some visible damages in some parts of the envelope. After a further culture at 30 °C for 2 h, the cell envelope remained slightly damaged (Figure 2C1), but to a lower extent that before recovery, indicating that *D. radiodurans* cells underwent some efficient repair mechanisms. These results show that heat stress may affects the structure of the bacterial cell wall but it is rather limited, having no significant effect on cell death, as revealed by fluorescence microscopy (Figure 2A2,B2,C2) showing that almost all cells remained alive.

### 2.2. Qualitative and Quantitative Analyses of the Differentially Expressed Genes (DEGs) in the Three Groups under Heat Stress

The phenotypic results presented above showed that the growth state of heat-treated cells almost reached that of the untreated cells after recovery for 2 h. We expected that a transcriptional analysis of these samples would provide a deeper understanding of the mechanisms responsible for adaptation of *D. radiodurans* to high temperature. Several DEGs representing different functional categories were selected for quantitative real-time PCR (qRT-PCR) analysis (Supplementary Figure S1). The expression profiles of the selected genes showed the same tendencies as those detected by RNA-Seq, indicating the good quality of the sequencing data.

**Figure 3:**
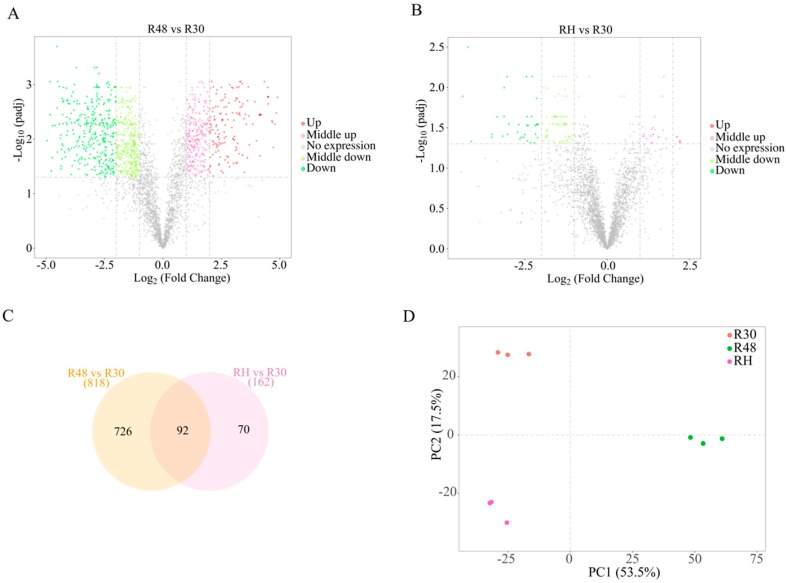
Differential expression levels among the three treatment groups. Each point represents a unigene. The *x*-axis represents the log_10_ values of the normalized expression level (FPKM) of the unigenes in each group. The red and green points indicate significant changes in the absolute value of log_2_ (the FPKM ratio in two groups) ≥ 1.5 and FDR ≤ 0.05, respectively; i.e., the red points indicate upregulated unigenes, and the green points indicate downregulated unigenes in the two groups, with the differential expression levels presented along the *X*-axis. The gray points indicate nonsignificant, differentially expressed unigenes. (**A**) R48 versus R30; (**B**) RH versus R30. (**C**) Venn diagram showing significant gene expression changes in response to heat. (**D**) Principal component analysis based on the FPKM expression values among the three treatment groups.

DEG analysis after fragments per kilobase of transcript per million mapped reads (FPKM) normalization yielded 818 and 162 genes with significantly different expression levels (FDR-corrected *p* < 0.05, ≥ 1.5 log_2_-fold change) in the pairwise comparisons of R48 versus R30 and RH versus R30 (Figure 3A,B; Supplementary Table S1). Among these genes, 316 were upregulated and 502 were downregulated (R48 versus R30) and 28 were upregulated and 134 were downregulated (RH versus R30). As shown in the Venn diagram (Figure 3C), 124 and 23 DEGs were unique to the pairwise comparisons of R48 versus R30 and RH versus R30. While investigating the gene expression of *D. radiodurans*, three replicates of R30, R48, and RH were used to create three distinct clusters via principal component analysis of the samples (Figure 3D). These data indicate that the DEGs have a very consistent and unique expression profile.

Regarding these significantly DEGs in response to heat stress, Table 1 presents a list of the top 30 most significantly up and downregulated-genes (R48 versus R30). Interestingly, we identified highly expressed genes (*dr_A0075*-*A0087*) with unknown functions. These genes are transcribed in the same transcription direction, and we hypothesized that they may be in the same operon and work together to cope with heat stress. In addition, some heat-induced genes that were reported in previous studies were also identified in our study (Table 1). We also identified many previously unreported genes that may be involved in this heat stress adaptation and wanted to further investigate the roles of these genes in the heat shock response.

### 2.3. Gene Ontology (GO) and Kyoto Encyclopedia of Gene and Genomes (KEGG) Analyses of the DEGs

To determine the functions of the DEGs, all of the DEGs were annotated to terms in the GO database. The most dominant subcategories were “oxidation-reduction process,” “cell,” “peptidase activity,” “cell part,” and “oxidoreductase activity” (Supplementary Figure S2; Supplementary Table S2). The top 20 most significantly enriched KEGG pathways identified via KEGG analysis are shown in Supplementary Figure S3. Several key molecular mechanisms are represented, including nitrogen metabolism, pyruvate metabolism, glycolysis, ribosome, oxidative phosphorylation, and propanoate metabolism. These results reveal that cells utilize numerous repair processes in response to high temperature.

### 2.4. Analysis of Heat-Related Genes with over Eight-Fold Higher Expressions in Response to Heat Stress

**Figure 4:**
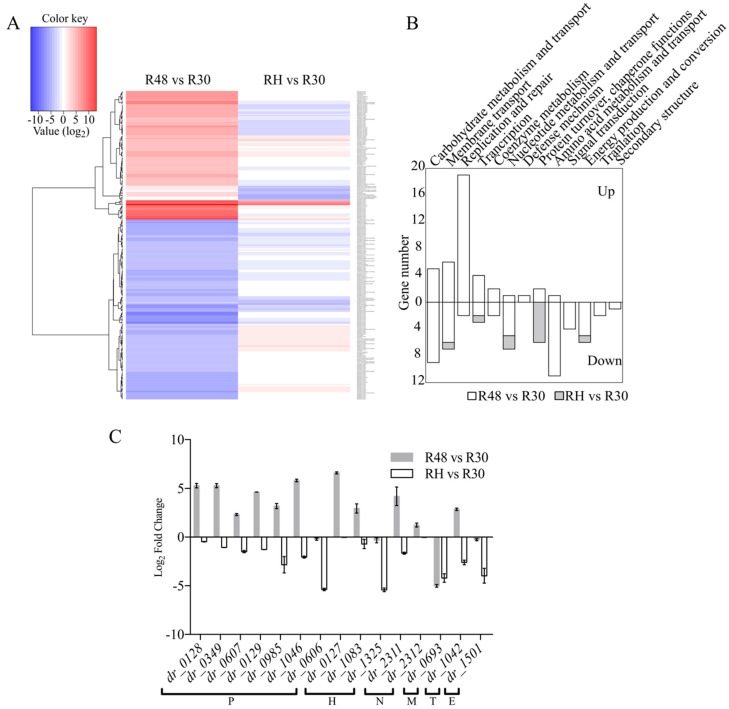
(**A**) Heatmap of the log2-fold changes (LFCs) among R30, R48, and RH. Included are 257 genes with fold changes >8. The dendrogram represents hierarchical clustering of the LFCs. (**B**) Likert chart of the KEGG functional categorizations of the differentially expressed genes between R48 versus R30 and RH versus R30. (**C**) qRT-PCR analysis of the high differentially expressed genes; P: protein turnover and chaperone functions, H: hypothetical protein, N: nucleotide metabolism and transport, M: membrane transport, T: transcription, E: energy production and conversion.

Comparisons among R30, R48, and RH revealed a total of 257 significantly regulated genes that exhibited at least an eight-fold difference in expression (Figure 4A). To obtain an overview of the functions that are altered during heat treatment and the recovery stage, 100 response genes (excluding hypothetical genes) were annotated with KEGG functional groups. Figure 4B presents the distributions within each category. A total of 19 genes were assigned to “Replication and repair.” The expressions of genes associated with “carbohydrate metabolism and transport” and “amino acid metabolism and transport” were greatly reduced at high temperature.

### 2.5. Function Analysis of the Novel, Potentially Heat-Related Genes

A total of 16 genes were differentially expressed (over eight-fold) in RH versus R30. Of these 16 genes, only one was upregulated, whereas 15 were downregulated (Table 2). These 15 genes showed at least an eight-fold downregulation in RH and were divided into the following categories: protein turnover and chaperone function (seven genes), hypothetical proteins (three genes), nucleotide metabolism and transport function (two genes), membrane transport (one gene), transcription (one gene), and energy production and conversion (one gene) (Figure 4C).

Three hypothetical genes (*dr_1325*, *dr_0127*, and *dr_1083*) were selected for further study. According to BLASTp, these three genes encode, respectively, for a predicted LysM peptidoglycan-binding protein (DR_1325), a predicted DNA-binding protein (DR_0127), and a hypothetical protein (DR_1083). Two of these genes, *dr_1325* and *dr_0127*, may be, respectively, involved in adaptation to heat stress through cell wall biogenesis and a DNA-binding protein, although their specific functions remain to be elucidated.

**Figure 5:**
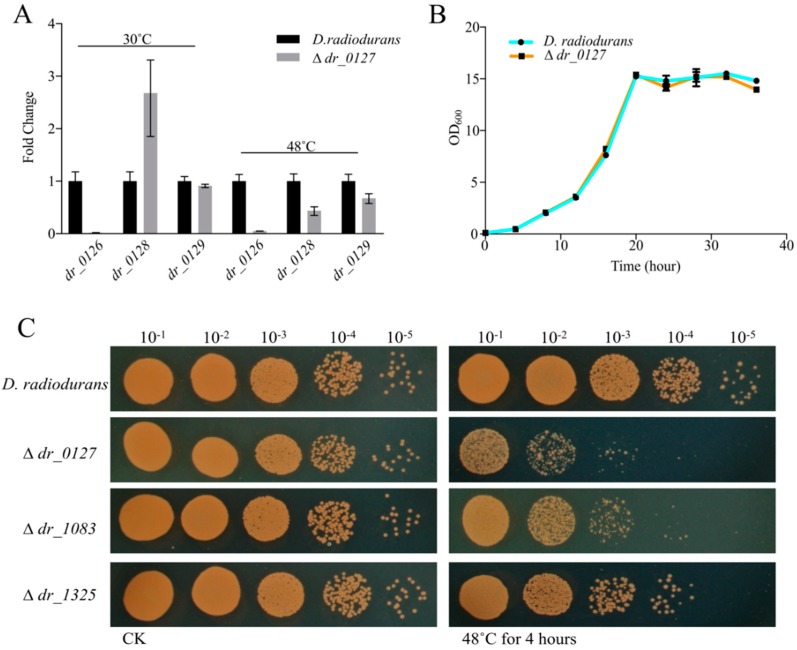
The effect of the *dr_0127* deletion on the expression of its flanking genes and survival phenotype plate assay upon heat stress. (**A**) The effect of the *dr_0127* deletion on the expression of its flanking genes (*dr_0126*, *dr_0128*, and *dr_0129*) under normal growth and heat stress conditions. The relative levels of transcripts are presented as the mean values ± standard deviations, calculated from three sets of independent experiments and normalized to levels in the wild-type strain. (**B**) Growth curves in TGY broth of wild type and ∆ *dr_0127*. The error bars represent the standard deviations of the measurements of three biological replicates. (**C**) Serial, 10-fold dilutions of OD-standardized *D. radiodurans* and three mutants (∆*dr_0127*, ∆*dr_1083*, and ∆*dr_1325*) spotted on TGY plates after exposure to 48 °C. CK, untreated culture control. All experiments were performed three times.

In order to further investigate the roles of those three genes *dr_0127*, *dr_1325*, and *dr_1083*, significantly upregulated in response to heat stress, disruption mutant strains were constructed and the insertion of the spectinomycin gene into each gene was confirmed by genomic PCR and sequencing (Supplementary Figure S4). To examine whether the *dr_0127* gene deletion had an effect on the expression of its flanking genes, we compared the expressions of *dr_0126*, *dr_0128*, and *dr_0129* in *D. radiodurans* wild type and Δ *dr_0127* by qRT-PCR. Figure 5A shows that the deletion of *dr_0127* caused a sharp decrease in the expression of *dr_0126*, increased the expression of *dr_0128* two-fold, and had no effect on *dr_0129* expression. At 48 °C, the deletion of *dr_0127* resulted in a decrease in the expression levels of these three genes. These results suggested that this deletion somehow affected the expression of the flanking genes expression under both normal and heat stress conditions, possibly because these genes work together in response to heat stress. In addition, the strain with the deletion of *dr_0127* showed no significant growth difference compared to the wild type under standard conditions (Figure 5B). A test for growth potential on TGY plate assays at a high temperature (48 °C) showed that the three mutant strains were more susceptible than the *D. radiodurans* wild-type, strongly suggesting that *dr_0127*, *dr_1083*, and *dr_1325* play key roles in the tolerance to heat stress (Figure 5C). Particular attention was devoted to *dr_0127* because the cognate mutant was the most severely impacted in terms of tolerance to heat shock (Figure 5C) and because it was the most differentially up-regulated upon heat stress (Figure 4C) among the 15 genes tested.

## 3. Discussion

In this study, we used RNA-Seq to investigate the mechanism of adaptation of *D. radiodurans* to heat stress. A sublethal high temperature is the best way to study heat stress genes [36]. Previous data have shown that 48 °C is the optimal temperature to study heat stress in *D. radiodurans* [9]. After the heat treatment, a large number of genes were differentially expressed to allow *D. radiodurans* to adapt to the external environment. Fluorescence electron microscopy showed that the heat treatment for 2 h did not cause death of *D. radiodurans* (Figure 2). This is consistent with previously reported results showing that almost all of the cells remained alive after 2 h of heat treatment [10]. To analyze the heat-related genes in detail, we performed recovery growth after the heat treatment. Our data show that after 2 h of recovery at 30 °C, the cells recovered normal life activities (Figure 1). Interestingly, the growth rate of *D. radiodurans* is approximately 2 h for one generation, a duration equivalent to our recovery time. We hypothesize that when *D. radiodurans* cells undergo self-repair after sublethal stress, it may take one generation for them to recover to prestress levels. The transcriptome data showed that most of the gene expression in RH was equivalent to that in R30, which confirmed that the 2 h of recovery allowed the intracellular homeostasis of the previous stage to be restored.

The cell wall is crucial for cellular function, especially for protecting cell physiological activity, as it separates the internal environment from the external environment. However, TEM showed that the *D. radiodurans* cell wall was slightly damaged after treatment at 48 °C treatment for 2 h (Figure 2B1). Although most of the cells remained alive (Figure 2B2), the high temperature damaged the integrity of the cell wall. Similarly, Stéphane Guyot et.al (2010) and Bożena Bruhn-Olszewska et al. (2018) reported that high temperatures can destroy the integrity of the cell wall in *E. coli* [37,38]. In this study, the TEM data showed that after the cells had recovered at 30 °C for 2 h, their cell walls showed partial repair (Figure 2C1). Complete cell wall repair would likely require additional time after the heat stress.

Chaperones and proteases function by preventing protein aggregation and facilitating the degradation of improperly folded proteins under heat stress conditions [39,40]. In the present study, heat-induced genes (including *groEL*, *lon1*, *lon2*, *dnaK*, and *hsp20*) were highly expressed in R48, with these genes having been well characterized in many organisms [20,41]. In *E. coli*, the aggregation of abnormal proteins stimulates the transcription of Lon protease to degrade damaged proteins [42]. There are two Lon homologs in *D. radiodurans* (DR_0349 and DR_1974), and deletion of these factors was observed to increase cell sensitivity to puromycin, indicating a reduced capacity to degrade abnormal proteins [43]. There are two sHSP members (IbpA and IbpB) in *E. coli*. These proteins were reported to be involved in the aggregation of proteins after heat stress [44,45]. Bepperling et al. reported that *D. radiodurans* also harbors IbpA (Hsp20) and IbpB (DR_1691), which can work in parallel and independently of each other to combat protein aggregation during stress [15]. In addition, *hsp20* was reported to confer tolerance to hydrogen peroxide [46]. These previous results and those of our transcriptomic analysis suggest that these proteases and chaperones likely play important roles in *D. radiodurans* during heat stress. Further analyses are needed to clarify the roles of chaperones and proteases in the heat tolerance of *D. radiodurans*.

The heat shock response of *D. radiodurans* illustrates the extensive diversity of gene regulation in this species. We identified genes with more than eight-fold differential expression between groups, with 15 genes in RH being downregulated by more than eight-fold relative to the expression observed in R30 (Figure 4). A subsequent investigation revealed that almost all these genes are involved in heat stress and that most are involved in protein turnover and chaperone function. Moreover, three of these genes are hypothetical genes (*dr_0127*, *dr_1083*, and *dr_1325*), among which one (*dr_0127*) was substantially upregulated in R48 (Figure 4). Annotation revealed that DR_0127 was predicted to be a DNA-binding protein. Genomic DNA packaging is mediated by a set of DNA-binding proteins that have major impacts on gene transcription and DNA replication [47,48,49]. The nucleoid of a bacterium is organized by DNA-binding proteins, especially under stress conditions; these proteins also protect the bacterial genome and regulate transcription to promote survival under stress conditions. Through alignment analyses, we observed that DR_0127 is highly conserved in other *Deinococcus* species, such as *D. wulumuqiensis*, *D. gobiensis*, *D. soli*, and *D. actinosclerus*. More interestingly, the upstream and downstream genes encode DnaJ, GrpE, and DnaK. The qRT-PCR data showed that deletion of *dr_0127* drastically decreased the expression of the downstream gene *dr_0126* (which encodes a DNA binding protein). Through the genome analysis, we predicted that these genes may share the same operon. In addition, we believe that the reduction in tolerance to heat stress may not only be due to the absence of *dr_0127* but also due to the dramatic downregulation of *dr_0126* expression. In future work, we will further clarify the exact relationship between *dr_0126* and *dr_0127* in response to heat stress by performing insertional mutagenesis. Deletion of *dr_0127* resulted in decreased expression of *dr_0126* (*dnaJ*), *dr_0128* (*grpE*), and *dr_0129 (dnaK)* relative to the wild-type strain under heat stress. DR_1027 is predicted to be a genus-specific DNA binding protein that we hypothesize may work together with DnaJ, GrpE, and DnaK in response to heat stress, although their mechanisms of action require further study. DR_1325 is predicted to be a protein containing a LysM peptidoglycan-binding domain, which may be involved in cell wall biogenesis. In addition, our phenotype experiments showed that deletion of DR_1325 resulted in sensitivity to heat stress compared to the wild type strain. *dr_1325* appears to be the first gene of the operon (*dr_1325-27*), and deletion of *dr_1325* also caused the upregulation of *dr_1326* and *dr_1327* (Supplementary Figure S5). The phenotype of the mutant strain resulting from the lack of the *dr_1325* may be caused by the changes of the transcription of the affected genes. Our results also show that heat stress damages the cell wall of *D. radiodurans* (Figure 2). We speculate that DR_1325 may regulate the response of the heat stress mechanism by participating in cell wall repair. That idea requires experimental support from further studies.

**Figure 6:**
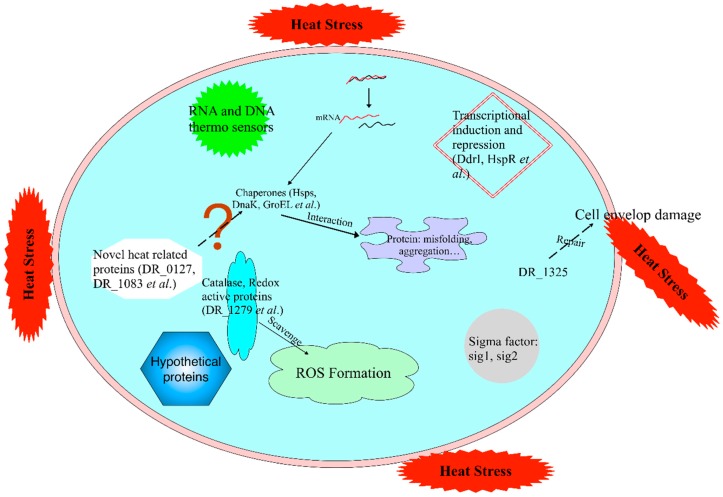
Molecular response of *D. radiodurans* under high temperature conditions. Chaperones, RNA and DNA thermosensors, transcription induction and repression, sigma factors, catalase and redox active proteins, and hypothetical proteins regulate the molecular mechanism of the heat stress response in cells. Chaperones mediate the correct folding of other polypeptides, such as hsp20 and DnaK; RNA and DNA thermosensors sense the temperature change; transcription induction and repression is involved in heat stress regulation, such as by HspR; sigma factors (sig1 and sig 2) that control heat-shock regulons have evolved to respond to protein misfolding; and catalase and redox active proteins scavenge the ROS caused by high temperature. Solid arrows and dashed lines represent the known and unknown mechanisms, respectively.

Taken together, the results of this study reveal a highly complex gene-expression response to heat stress in *D. radiodurans* that involves numerous key genes related to various cell processes. Chief among these genes are those encoding chaperones, heat shock proteins, proteases, and posttranscriptional regulatory proteins (Figure 6). Our study also revealed novel, potentially important features exhibited by *D. radiodurans* in response to heat stress, such as the substantial recovery of RH cells compared with R30 cells, confirming that *D. radiodurans* also has a strong self-repair ability under heat stress. By analyzing the transcriptome data of the three groups, some new heat-related genes were discovered and verified, which expand the heat stress-related gene family in *D. radiodurans*. Further metabolome analyses and gene knockout experiments, particularly those targeting the novel genes, will enhance our understanding of the molecular mechanisms underlying heat stress in this species.

## 4. Materials and Methods

### 4.1. Strain and Growth Conditions

*D. radiodurans* was obtained from the China General Microbiological Culture Collection Center (CGMCC 1.633, Beijing, China). *D. radiodurans* was cultured at 30 °C in TGY medium (1% tryptone, 0.5% yeast extract, and 0.1% glucose) with shaking at 220 rpm/min.

### 4.2. Heat Stress Treatment of D. radiodurans and Recovery Conditions

Bacterial cells were pre-cultured in TGY to an OD_600_ = 2, were harvested by centrifugation at 7000× *g* for 3 min, washed twice in sterile phosphate-buffered saline (PBS, 0.02% KH_2_PO_4_, 0.29% Na_2_HPO_4_·12H_2_O, 0.8% NaCl, 0.02% KCl, pH 7.5), and resuspended in fresh TGY broth to the same cell density. For heat stress, cells were incubated at 48 °C for 2 h. They were then collected by centrifugation and resuspended to the same OD in TGY pre-conditioned at 30 °C. Bacteria were transferred to a second incubator pre-set at 30 °C and grown as a recovery culture for 0.5, 1, 1.5, or 2 h. A total of 100 μL was collected for dilution and plating onto solid TGY medium to calculate the number of colony-forming units (CFU). Cells that did not receive the heat treatment (2 h of growth at 30 °C) served as controls.

For transcriptomics, cells were treated at 48 °C for 2 h and then divided into two samples (1 and 2). Cells from sample 1 that were harvested by centrifugation at 12,000× *g* for 3 min and stored at −80 °C served as heat-treated group (R48). Cells from sample 2 that were centrifuged at 7000× *g* for 3 min, washed twice in PBS, and transferred to fresh TGY medium for recovery growth at 30 °C for 2 h served as the recovery group (RH). The restored cells were harvested by centrifugation at 12,000× *g* for 3 min and stored at −80 °C. Untreated *D. radiodurans* bacteria were used as the control (R30). All assays were performed in triplicate.

### 4.3. TEM and Fluorescence Assay

For TEM analysis, cells grown to OD_600_ = 2.0 were washed twice with PBS. The cells in the three treatment groups were collected and fixed overnight with 2.5% glutaraldehyde at 4 °C and then embedded in 2% agarose after centrifugation at 3000× *g*. Thin sections of the samples were stained with uranyl acetate for 15 min and observed using a Hitachi H-7650 transmission electron microscope (Hitach, Tokyo, Japan).

Cell viability after heat treatment was studied by staining with specific fluorochromes followed by epifluorescence microscopy. Cells were stained using a BacLight^TM^ RedoxSensor^TM^ Green Vitality Kit (ThermoFisher, MA, USA) containing PI and RSG. *D. radiodurans* cells were washed with PBS. This kit is convenient and easy-to-use for monitoring the viability of bacterial populations as a function of cell membrane integrity. Cells with a compromised membrane, which are considered dead or dying, stain red (PI), whereas cells with an intact membrane stain green (RSG). A fluorescence assay was performed using a 100× oil immersion lens with a *Nikon* T*i*2 inverted fluorescence microscope and processed with the NIS-Elements (*Nikon*, Tokyo, Japan).

### 4.4. Total RNA Extraction, complementary DNA (cDNA) Library Preparation, and Sequencing

Total cellular RNA was extracted from *D. radiodurans* using TRIzol reagent (Invitrogen, Thermo Fisher, MA, USA), Lysing Matrix Tubes (MP Bio, CA, USA), and the PureLink RNA Mini Kit (Invitrogen, Thermo Fisher, MA, USA) following the manufacturer’s instructions. RNA purity was assessed using a NanoDrop^®^ spectrophotometer (Thermo Fisher, MA, USA). The RNA concentration was measured using a Qubit^®^ RNA Assay Kit and a Qubit^®^ 3.0 Fluorometer (Life Technologies, CA, USA). RNA integrity (RIN) was assessed using an RNA Nano 6000 Assay Kit and the Bioanalyzer 2100 system (Agilent Technologies, CA, USA), RIN > 9.5.

A total of 1 μg of high-quality RNA per sample was used as the input material for library preparation. Sequencing libraries were generated using a VAHTS Total RNA-Seq Library Prep Kit for Illumina^®^ (Vazyme, NR603), following the manufacturer’s recommendations. Following purification, the RNA was fragmented into small pieces using divalent cations under an elevated temperature. The cleaved RNA fragments were copied into first-strand cDNA using reverse transcriptase and random primers. Strand specificity was achieved by replacing dTTP with dUTP in 2nd strand marking buffer, followed by second-strand cDNA synthesis using DNA Polymerase I and RNase H. Then, the cDNA fragments were end-repaired with the addition of a single “A” base at the 3′-end of each strand and subsequently ligated to special sequencing adapters (Vazyme, N803). PCR was performed, and the products were purified. The library concentration was measured using a Qubit^®^ RNA Assay Kit in Qubit^®^ 3.0 for preliminary quantification. The sizes of the inserted fragments were assessed using the Agilent Bioanalyzer 2100 system, and high-quality insert fragments were accurately amplified using qPCR with the StepOne Plus Real-Time PCR system (ABI, USA). Clustering of the index-coded samples was performed on the cBot Cluster Generation System (Illumina, USA) according to the manufacturer’s instructions. Then the well-prepared library was sequenced using the Illumina HiSeq X Ten platform with a 150-bp paired-end module. All samples were sequenced three times.

### 4.5. Assembly and Functional Enrichment Analyses of DEGs

Clean reads with an average length of 150 bp were achieved after removing contaminated, poly-N and low-quality sequences from the raw reads. On average, 30.6 million transcripts were mapped to the reference genome with sample sizes ranging between 28.3 and 40.2 million reads (Supplementary Table S3). Pearson correlation analysis showed that the overall expression levels among the three biological replicates of each group were highly similar (R^2^ > 0.92; Supplementary Figures S6 and S7), indicating that the RNA-Seq data were suitable for pairwise statistical comparisons. The reference genome and gene model annotation files of D. radiodurans were directly collected from the genome website (https://www.ncbi.nlm.nih.gov/genome/1020?genome_assembly_id=300483). The reference genome index was built using Bowtie2 (v2.2.9) [50], and paired-end clean reads were aligned to the reference genome using TopHat (v2.1.1) [51]. The mapped reads of each sample were assembled using Cufflinks (v2.2.1) [52] with a reference-based approach. Cufflinks uses a probabilistic model to simultaneously assemble and quantify the expression levels of a minimal set of isoforms, which provides a maximum likelihood explanation of the expression data for a given locus. Cuffdiff (v1.3.0) [52] was used to calculate the FPKMs of the coding genes in each sample. The gene FPKMs were computed by summing the FPKMs of the transcripts in each gene group. Cuffdiff (v2.2.1) [52] provides statistical routines for determining differential expression in a digital transcript or gene expression dataset using a model based on a negative binomial distribution. Genes with corrected *p*-values less than 0.05 and absolute log_2_ values (fold changes) >1.5 were considered significant DEGs.

### 4.6. GO and KEGG Enrichment Analysis

GO is a standardized system for classifying gene functionality and provides a dynamically-updated controlled vocabulary for fully characterizing gene properties and products in organisms. GO analysis is classified into three domains: biological processes, cellular components, and molecular functions of gene products. GO enrichment analysis of the DEGs was performed with the Perl module (GO::TermFinder) [53]. GO terms with a corrected *p*-values less than 0.05 were considered to be significantly enriched among the DEGs. In vivo, different genes coordinate with each other to perform their biological functions, and pathway-based analysis is helpful to understand the biological functions of gene interactions in various pathways. KEGG is a major public database containing manually-drawn pathway maps representing knowledge of molecular interactions and reaction networks. R functions (phyper and *q*-value) were used to test for the statistical enrichment of the DEGs among the KEGG pathways. KEGG pathways with corrected *p*-values less than 0.05 were considered to be significantly enriched for the DEGs.

### 4.7. qRT-PCR Validation

We randomly selected several genes with or without detectably differential expression for subsequent qRT-PCR analysis to verify the quality of the sequencing data. Total RNA was extracted as described for the cDNA library preparation, and RNA was used for cDNA synthesis using a PrimeScript^TM^RT reagent kit with gDNA Eraser (TaKaRa) as described in the manufacturer’s protocol. Subsequently, qRT-PCR was performed using ChamQ SYBR qPCR Master Mix (Vazyme Biotech Co., Ltd., China) on a 7500 Fast Real-time PCR System (Applied Biosystems, USA). The primers are listed in Supplementary Table S4. The 16S rRNA gene was used as the endogenous reference control to normalize differences in total RNA quantity, and relative gene expression was quantified by the 2^−ΔΔ*C*T^ method. Three biological replicates for each condition were conducted.

### 4.8. Construction of Gene Deletion Mutant Strains and Heat Stress Phenotype Assays

Mutant strains lacking *dr_0127*, *dr_1325*, and *dr_1083* were constructed by fusion PCR recombination of a spectinomycin resistance cassette into the genome as previously described [54]. Briefly, fusion PCR products for the *dr_0127*, *dr_1325*, and *dr_1083* deletions were constructed in two steps. In the first step, three different PCRs were used to generate fragments complementary to the spectinomycin-resistance gene from the plasmid pKatAAD2 (896 bp) and the upstream and downstream regions (500 bp each) of the *dr_0127*, *dr_1325*, and *dr_1083* sequences using the appropriate primer pairs (Supplementary Table S4). In the second step, the upstream, spectinomycin-resistance gene and downstream fragments were annealed at their overlapping regions and PCR amplified as a single fragment using the outer primers (1896 bp). The resulting PCR fragment was directly transformed into *D. radiodurans*. Colonies resistant to spectinomycin (340 μg/mL) were selected, and these mutants were subsequently verified by PCR and DNA sequencing, and named Δ *dr_0127*, Δ *dr_1325*, and Δ *dr_1083*.

Cells were grown in TGY medium with the appropriate antibiotics to OD_600_ = 2 at 30 °C and were then shifted to 48 °C for 4 h. Subsequently, 100 μL of the cell suspension was aliquoted into 900 μL of PBS, after which 10-fold serial dilutions were made for all the strains, and 8 µL of each dilution was spotted onto TGY agar plates. These plates were incubated at 30 °C for 3 days before colony growth was observed and calculated. All assays were performed in triplicate.

### 4.9. Statistical Analysis

All experiments were repeated at least three times with identical or similar results. The mean values from the individual experiments were expressed as averages ± standard deviations (SD). A *p*-value < 0.05 was considered to be significant. R studio and GraphPad Prism 7.0 software were used for the analysis.

Data Accessibility: The sequences were uploaded and deposited in the National Center for Biotechnology Information (NCBI) Sequence Read Archive (SRA) under the accession numbers SRR9851810 to SRR9851818.

## Figures and Tables

**Table 1:** The top 30 most upregulated and downregulated genes when exposed to heat stress (R48 versus R30).

Gene ID	Log2 (Fold Change)	*p* Value	Function Description	References
DR_A0081	13.0548	0.02115134	Hypothetical protein	
DR_A0078	9.3231	0.01952321	Hypothetical protein	
**DR_2307**	8.5243	1.16 × 10^−5^	Multidrug-efflux transporter, putative	[10]
DR_0518	8.3853	0.00804687	Hypothetical protein	
DR_0524	8.1827	0.00604411	Hypothetical protein	
DR_A0082	7.8581	0.02528407	Hypothetical protein	
DR_A0079	7.745	0.0212175	Hypothetical protein	
DR_A0080	7.6759	0.0183724	Hypothetical protein	
DR_A0083	7.4529	0.02027402	Hypothetical protein	
DR_A0086	7.3763	0.01788636	Hypothetical protein	
DR_0519	7.3405	0.00011836	Hypothetical protein	
DR_A0101	7.2857	0.0161855	Hypothetical protein	
DR_A0077	7.1326	0.01621666	Hypothetical protein	
DR_A0075	6.9708	0.04170709	Transposase, putative	
DR_A0087	6.5568	0.01613077	Hypothetical protein	
DR_B0072	6.0839	5.39 × 10^−6^	Salicylate monooxygenase-related protein	
DR_B0074	6.0164	7.87 × 10^−7^	1-Phosphofructokinase	
DR_B0073	5.8393	1.1 × 10^−5^	PTS system, fructose-specific IIBC component	
DR_A0085	5.833	0.02305785	Hypothetical protein	
DR_A0211	5.5236	4.38 × 10^−6^	Transcriptional regulator, GntR family	
DR_C0023	5.1883	2.24 × 10^−5^	Hypothetical protein	
DR_A0076	5.1069	0.02545438	ATP-dependent target DNA activator	
DR_B0141	5.0516	9 × 10^−5^	HicB-related protein	
DR_B0142	4.9077	5.11 × 10^−5^	Hypothetical protein	
DR_0422	4.8592	0.00024562	Trans-aconitate 2-methyltransferase	
**DR_A0182**	4.799	0.00068635	Hypothetical protein	[13]
DR_0516	4.7289	0.00382339	Hypothetical protein	
**DR_2374**	4.6897	0.0005564	Ribonucleoside-diphosphate reductase-related protein	[13]
DR_0532	4.6735	0.00051046	Hypothetical protein	
DR_0423	4.5152	0.00011999	Hypothetical protein	
**DR_A0364**	−4.3096	5.93 × 10^−5^	Oxidoreductase, short-chain dehydrogenase/reductase family	[13]
DR_0201	−4.321	0.00785386	Hypothetical protein	
**DR_0392**	−4.3342	0.00434331	Hypothetical protein	[10]
DR_0334	−4.3572	0.01241591	Lipase, putative	
DR_B0038	−4.3573	0.01463299	Hypothetical protein	
DR_A0352	−4.3662	0.00155047	Methyl-accepting chemotaxis protein	
DR_2240	−4.3942	0.00195473	Hypothetical protein	
**DR_2527**	−4.4273	0.00068251	Hypothetical protein	[10]
**DR_A0233**	−4.4361	1.05 × 10^−5^	Oxidoreductase, iron-sulfur subunit	[13]
DR_1987	−4.4998	3.18 × 10^−6^	Hypothetical protein	
**DR_A0231**	−4.5169	0.00510755	Oxidoreductase	[13]
DR_1778	−4.5277	0.00016505	3-Isopropylmalate dehydratase, large subunit	
**DR_2263**	−4.5338	6.47 × 10^−8^	DNA-binding stress response protein, Dps family	[10]
DR_2563	−4.6247	0.00080366	Hypothetical protein	
**DR_1712**	−4.7284	0.00739225	Extracellular solute-binding protein, family 5	[13]
DR_A0232	−4.7289	0.00081206	Oxidoreductase	
DR_1277	−4.7754	0.01673083	ABC transporter, periplasmic substrate-binding protein, putative	
DR_1711	−4.8264	0.00028517	N-Acyl-L-amino acid amidohydrolase	
DR_1067	−4.8305	5.2 × 10^−6^	Hypothetical protein	
DR_0791	−4.9522	0.00226901	Chloride peroxidase, putative	
DR_0644	−5.4271	0.00078552	Hypothetical protein	
DR_2560	−5.4566	0.0002672	Hypothetical protein	
**DR_1315**	−5.4743	0.00327873	Hypothetical protein	[10,13]
DR_0105	−5.4893	1.54 × 10^−6^	Hypothetical protein	
DR_1066	−5.6713	0.02719424	Hypothetical protein	
DR_1483	−5.8009	0.00071264	Hypothetical protein	
DR_1790	−5.8218	1.62 × 10^−6^	Yellow-related protein	
DR_0465	−5.8342	0.00052829	Conserved hypothetical protein	
**DR_1314**	−6.1749	0.00281391	Conserved hypothetical protein	[10]
DR_0891	−6.4635	0.00113242	DNA-binding response regulator	

Note: the previously reported genes involved in heat stress are indicated in bold font.

**Table 2:** Descriptions of the 16 genes with altered expression in cells recovered from heat stress compared with non-stressed cells (RH versus R30).

Gene ID	Gene Name	Functions	Log2(Fold Change)	*p* Value
DR_A0101		Hypothetical protein	6.7807	0.00190534
DR_0128	*grpE*	Protein turnover and chaperone function	−3.1812	0.00160996
DR_0349	*lon*	Protein turnover and chaperone function	−3.5087	0.00188947
DR_0607	*groEL*	Protein turnover and chaperone function	−3.5102	0.00128474
DR_0129	*dnaK*	Protein turnover and chaperone function	−3.9169	0.04542787
DR_0985		Protein turnover and chaperone function	−4.1346	0.00249103
DR_1046	*clpB*	Protein turnover and chaperone function	−4.4467	0.01832036
DR_0606	*groES*	Protein turnover and chaperone function	−3.3489	0.00035202
DR_0127		DNA-binding protein	−3.0322	0.00135957
DR_1083		Hypothetical protein	−3.1333	4.23 × 10^−5^
DR_1325		LysM peptidoglycan-binding protein	−3.3623	0.00886153
DR_2311		Nucleotide metabolism and transport function	−4.3949	9.48 × 10^−5^
DR_2312		Nucleotide metabolism and transport function	−4.2404	1.03 × 10^−6^
DR_1501		Energy production and conversion	−3.1352	0.00033852
DR_0693		Membrane transport	−3.7209	0.00754623
DR_1042	*padR*	Transcription	−3.0467	1.6 × 10^−5^

## Supplementary Materials

Supplementary materials can be found at https://www.mdpi.com/1422-0067/20/22/5603/s1.
