# Supplementary material for: RNA-Seq-Based Comparative Transcriptome Analysis Highlights New Features of the Heat-Stress Response in the Extremophilic Bacterium Deinococcus radiodurans
Source: Int J Mol Sci. 2019 Nov 9;20(22):5603. doi: 10.3390/ijms20225603 (PMC6888292; doi:10.3390/ijms20225603)
Supplement: Supplementary file 1 [file ijms-20-05603-s001.zip › Supplementary files/Supplementary Figures and tables.docx]

Supplementary Material

# **RNA-Seq-based** **comparative transcriptome analysis highlights new features of the heat stress response in the extremophilic bacterium *Deinococcus radiodurans***

Dong Xue, Wenzheng Liu, Yun Chen, Yingying Liu, Jiahui Han, Xiuxiu Geng, Jiang Li, Shijie Jiang, Zhengfu Zhou, Wei Zhang, Min Chen, Min Lin, Marc Ongena^*^, Jin Wang^*^

*** Correspondence:** Marc Ongena: Marc.Ongena@ulg.ac.be; Jin Wang: wangjin@caas.cn

# 1 Supplementary Figures and Tables

## 1.1 Supplementary Figures

**Supplementary Figure S1.** Validation of the RNA-Seq data using qRT-PCR. Black bars represent the RNA-Seq data, and white bars represent mean log_2_ values (fold changes). Data are presented as the mean ± SD values of triplicate reactions for each gene transcript.

**Supplementary Figure S2.** Gene ontology (GO) enrichment analysis of differentially expressed genes in *D. radiodurans* with and without heat stress. The 30 most enriched GO terms are shown. (A: R48 vs R30, B: RH vs R30)

**Supplementary Figure S3.** Top 20 KEGG biological pathway classification histograms for annotated unigenes. (A: R48 vs R30, B: RH vs R30)

**Supporting Figure S4.** Construction and verification of the three mutants. (A) Schematic representation of the mutant generated by replacing the *dr_0127*, *dr_1325*, or *dr_1083* region with the spectinomycin resistance gene *aadA*. (B) PCR verification of the Δdr_1325 mutant. Lane M: Trans2K PlusII DNA marker; lanes 1: PCR products amplified from *D. radiodurans* WT using *aadA* primers; lanes 2: PCR products amplified from the Δdr_1325 mutant using *aadA* primers; lanes 3: PCR products amplified from the Δ*dr_1325* mutant using upstream and downstream gene primers; and lanes 4: PCR products amplified from *D. radiodurans* WT using upstream and downstream gene primers. (C) PCR verification of the Δ*dr_0127* mutant. Lane M: Trans2K PlusII DNA marker; lanes 5: PCR products amplified from *D. radiodurans* WT using *aadA* primers; lanes 6: PCR products amplified from the Δ*dr_0127* mutant using *aadA* primers; lanes 7: PCR products amplified from the Δ*dr_0127* mutant using upstream and downstream gene primers; and lanes 8: PCR products amplified from *D. radiodurans* WT using upstream and downstream gene primers. (D) PCR verification of the Δ*dr_1083* mutant. Lane M: Trans2K PlusII DNA marker; lanes 9: PCR products amplified from *D. radiodurans* WT using *aadA* primers; lanes 10: PCR products amplified from the Δ*dr_1083* mutant using *aadA* primers; lanes 11: PCR products amplified from the Δ*dr_1083* mutant using upstream and downstream gene primers; and lanes 12: PCR products amplified from *D. radiodurans* WT using upstream and downstream gene primers.

**Supplementary Figure** **S**5 Effect of the *dr_1325* deletion on the expression of its downstream genes (*dr1326* and *dr1327*) under normal growth conditions. The relative levels of the transcripts are presented as the mean values ± standard deviation, calculated from three sets of independent experiments and normalized to levels in the WT strain.

**Supplementary Figure S.** Pearson correlation analysis among 9 samples

**Supplementary Figure S7.** Cluster analysis of differentially expressed genes in 9 samples under heat stress

## 1.2 Supplementary Tables

**Supplementary Table S3.** Summary of the control and heat stress transcriptome sequencing data

| Name | Read length | Raw reads | Clean reads | Raw_Q20 | Clean_Q20 | | Total mapped reads |
| --- | --- | --- | --- | --- | --- | --- | --- |
| R30-1 | 150 bp | 40243726 | 35593566 | 96.47% | 96.34% | 82.3% | |
| R30-2 | 150 bp | 36659576 | 32317906 | 96.75% | 96.64% | 84.9% | |
| R30-3 | 150 bp | 36686154 | 32648744 | 96.13% | 95.98% | 85.2% | |
| R48-1 | 150 bp | 30941592 | 27268952 | 96.45% | 96.29% | 83.9% | |
| R48-2 | 150 bp | 28375830 | 25064318 | 96.34% | 96.16% | 80.7% | |
| R48-3 | 150 bp | 29987026 | 26644728 | 96.25% | 96.09% | 85.3% | |
| RH-1 | 150 bp | 37883232 | 32560522 | 96.25% | 96.11% | 88.0% | |
| RH-2 | 150 bp | 39150736 | 34891584 | 96.47% | 96.36% | 89.0% | |
| RH-3 | 150 bp | 32387858 | 28419606 | 96.22% | 96.08% | 82.4% | |

**Supplementary Table S4.** List of primers used for this study

| Gene name | Primers (5’ to 3’) | Purpose |
| --- | --- | --- |
| DR_0606 (groES) | F: GCGACCGCGTTCTGGTTG  R: GTACTTGGCGAAGTACACG | qRT-PCR |
| DR_1114 (hsp20) | F: GGCCTCGAACTGACCTTGGACATTC  R: AAAGGTGCCGTAGGCGCGCTCGACA | qRT-PCR |
| DR_1655 | F: CTGCGCCACCTGCTGCACG  R: CGTTCACGATGAGCTTGGC | qRT-PCR |
| DR_1738 | F: GAGTGTGCCGTCCCAGGTG  R: GAGGTCAGACAGTGCCGTGTC | qRT-PCR |
| DR_0128 (grpE) | F: GCCGGACACCGAAGCCGAC  R: CCAGCTCGTCCACGCGCTCGAGC | qRT-PCR |
| DR_0127 | F: GCACGACGAGTACGGGTG  R: CGTGAAGTCCAGCGCCTGC | qRT-PCR |
| DR_0349 | F: CGACGGCACCTTGCAGATGC  R: CTTGGAGTTGATGGTCTGC | qRT-PCR |
| DR_1046 (ClpB) | F: GAACCCTGCTCGACAACGAC  R: GCGAGCGTGTCGGCCTTCTG | qRT-PCR |
| DR_0129 (dnaK) | F: GTCAGGCTGCGCTCAAC  R: CGTCGCTCACCAGCTTGCGC | qRT-PCR |
| DR_0607 (groEL) | F: GAGCTGGAGGACAAGCTG  R: GCCACGGCCTTGTCGATGC | qRT-PCR |
| DR_1083 | F: tggctttgctgctgttgctg  R: tggcggtgcccgtgaccgtc | qRT-PCR |
| DR_0985 | F: tgaaagcgacggcaaactcg  R: aggtaacggctcacgtcctc | qRT-PCR |
| DR_1042 | F: cctcaaatctggcacggtg  R: caatccagtcggccttgctc | qRT-PCR |
| DR_1501 | F: acgctgatgtgcgcgcttgc  R: agccctcgtcgttgagctg | qRT-PCR |
| DR_1325 | F: tcctgaccaccctgatcttg  R: tcgggcgaggcgaggttgttg | qRT-PCR |
| DR_2311 | F: tctggacattggcctgac  R: agcacgcgggcggcctcg | qRT-PCR |
| DR_2312 | F: cgacgagagtcacgagttgc  R: tgagaggtcagggacgacg | qRT-PCR |
| DR_0693 | F: ttcttttacggcggcctgac  R: cgaacatggcctgaaacgag | qRT-PCR |
| DR0126 (dnaJ) | F: AGGACTACTACGACGTGCTCG  R: TGGCCGTACTGGTCGTACAG | qRT-PCR |
| DR_r06 (16S) | F: ATTCCTGGTGTAGCGGTG  R: CATCGTTTAGGGTGTGGAC | qRT-PCR |
| DR_0127-up | F: TGGAACCCGCCGCCCATGCCG  R: CAAGGGCCCTCGGTCTCCATGCGACCACACAAGGAAAGGAGGTTTATG | *DR_0127* mutant construction |
| DR_0127-middle | F: CCTCCTTTCCTTGTGTGGTCGCATGGAGACCGAGGGCCCTTGACATTG  R: GCTGATTATGTTCATGTTGATTTATTTGCCGACTACCTTGGTGATCTC |  |
| DR_0127-down | F: ACCAAGGTAGTCGGCAAATAAATCAACATGAACATAATCAGCCAGAGAG  R: ATTCAGGTCGTTGCTGGTGACGAAG |  |
| DR_1325-up | F: CGTCTTCATGGGAGCTTGCTGTG  R: CAAGGGCCCTCGGTCTCCATGCGGGCTGTCTCTCAGGGGAAGGCACTC | *DR_1325* mutant construction |
| DR_1325-middle | F: CTTCCCCTGAGAGACAGCCCGCATGGAGACCGAGGGCCCTTGACATTG  R: CCCGCTCCGCGCTCAGTGCGCTTATTTGCCGACTACCTTGGTGATCTC |  |
| DR_1325-down | F: ACCAAGGTAGTCGGCAAATAAGCGCACTGAGCGCGGAGCGGGTCCGAAG  R: GTGCTCACCGCGTCCTGGCCGAG |  |
| DR_1083-up | F: CGCCGAGGCCGAGGTCAATGC  R: CAAGGGCCCTCGGTCTCCATGGTTGCACCCTAAAACCCCCGACACGGA | *DR_1083* mutant construction |
| DR_1083-middle | F: TCGGGGGTTTTAGGGTGCAACCATGGAGACCGAGGGCCCTTGACATTG  R: GTTCGCATGTCCTCATCAGGCTTATTTGCCGACTACCTTGGTGATCTC |  |
| DR_1083-down | F: ACCAAGGTAGTCGGCAAATAAGCCTGATGAGGACATGCGAACGCTCGT  R: TCCAGGGGCGGGCGGCGTACATG |  |
